# Competence and Confidence of Healthcare Professionals in Using Clozapine: A Qualitative Systematic Review and Thematic Synthesis

**DOI:** 10.1192/bjo.2025.10222

**Published:** 2025-06-20

**Authors:** Agostina Secchi

**Affiliations:** Kent and Medway NHS Partnership Trust, Maidstone, United Kingdom. University of Kent, Canterbury, United Kingdom

## Abstract

**Aims:** Clozapine is the only licensed medication for treatment-resistant schizophrenia although it is underused. Healthcare providers’ (HCP) competence and confidence appear to have an effect on clozapine underutilisation. This review aims to synthesize the most pertinent literature examining the factors influencing HCP competence and confidence in the management of clozapine and how these factors influence variation in prescribing practice.

**Methods:** A review of the literature focusing on these elements was conducted. The **P**opulation, **C**ontext, **O**utcome **(PCO)** framework was adopted to support the literature search. The databases Medline, Psychinfo, Scopus, Cinahl, Pubmed, Embase, British Library, Ethos e-thesis, Google Scholar, Dart Europe e-thesis were consulted; the search was completed in January 2025. Screening, selection, data extraction and quality assessment were conducted independently by 2 researchers. Thematic analysis was used to investigate and compare the data emerging from the studies.

**Results:** Thirty-four articles were included in the review. Six themes were identified: attitude toward and knowledge about clozapine, misconceptions (regarding side effects, monitoring and co-morbidities), guidance, education, training and experience. Clinicians self-reported as competent with guidelines, yet they expressed less confidence in their ability to adhere to them and were uncertain about managing side effects. Lack of education, training and insufficient exposure to clozapine management were significant factors impacting competence and confidence resulting in clozapine underuse. Few studies involving non-medical professionals highlighted a general lack of education and training related to clozapine use.

**Conclusion:** Deficiencies in knowledge and experience were identified among professionals. However, the studies included in this review were lacking in the involvement of non-medical professionals. Given their crucial role in managing side effects and educating patients and carers, it is evident that their inclusion in future research is imperative.

